# Lineage tracing of mutant granulosa cells reveals in vivo protective mechanisms that prevent granulosa cell tumorigenesis

**DOI:** 10.1038/s41418-023-01132-1

**Published:** 2023-02-23

**Authors:** Shudong Niu, Kaixin Cheng, Longzhong Jia, Jing Liang, Lu Mu, Yibo Wang, Xuebing Yang, Chen Yang, Yan Zhang, Chao Wang, Lijun Huang, Huarong Wang, Shuang Zhang, Hua Zhang

**Affiliations:** 1 State Key Laboratory of Agrobiotechnology, College of Biological Sciences, China Agricultural University, Beijing, 100193 China; 2 Department of Obstetrics and Gynecology, Guangdong Provincial Key Laboratory of Major Obstetric Diseases, The Third Affiliated Hospital of Guangzhou Medical University, Guangzhou, China

**Keywords:** Cancer models, Predictive markers

## Abstract

Ovarian granulosa cell tumors (GCTs) originate from granulosa cells (GCs) and represent the most common sex cord-stromal tumor in humans. However, the developmental regulations and molecular mechanisms underlying their etiology are largely unknown. In the current study, we combined a multi-fluorescent reporter mouse model with a conditional knockout mouse model, in which the tumor suppressor genes *Pten* and *p27* were deleted in GCs, to perform cell lineage tracing of mutant GCs. We found that only 30% of ovaries with substantial mutant GCs developed into GCTs that derived from a single mutant GC. In-depth molecular analysis of the process of tumorigenesis demonstrated that up-regulation of immune evasion genes *Cd24a* and *Cd47* led, in part, to the transition of mutant GCs to GCTs. Therefore, treatment with the *Cd47* inhibitor RRX-001 was tested and found to efficiently suppress the growth of GCTs in vivo. Together, our study has revealed an immune evasion mechanism via CD24/CD47 upregulation to GCT formation, shedding light on the future potential clinical therapies for GCTs.

## Introduction

In the ovary, granulosa cells (GCs) produce sex hormones and support the development of oocytes [1, 2]. To achieve these functions, GCs are the most active cell lineage in the ovaries in terms of differentiation and proliferation. Granulosa cell tumors (GCTs), which derive from GCs, are the most common sex cord-stromal tumor; however, they constitute less than 5% of all ovarian tumors [3]. Although the incidence of GCTs is relatively low, there is a high chance of recurrence and development of life-threatening advanced tumors [4, 5]. Given that relapsed GCTs do not respond well to current standard chemotherapy, novel targeted therapeutics are urgently needed.

Forkhead box protein L2 (FOXL2), a member of the forkhead-winged helix family, is highly conserved and required for the maintenance of GCs [6, 7]. A somatic point mutation, 402 C → G (C134W), in FOXL2 has been identified in almost all human adult types of GCTs [8]. However, due to the difficulties associated with in vivo studies on human patient, the pathogenesis of GCTs at the developmental and molecular levels has yet to be elucidated. In addition, the utilization of genetically modified mouse models has led to the identification of several genes reportedly involved in GCT formation [9–11]. These genes include *Inha*, *Smad1*, and *Smad5* in the TGFβ pathway or constitutively active TGFβ signaling [12–14], and *Foxo1*, *Foxo3*, and *Pten* in the PI3K pathway. Interestingly, all the studied mouse models exhibited low GCT incidence in mutant females, which is consistent with the pathological characteristics of human GCTs. These studies shed light on the molecular mechanisms regulating GCT tumorigenesis and implied that the development of GCTs from mutant GCs is a complicated process. However, due to the lack of appropriate strategies for tracing the development of mutant GCs in vivo, the GCT formation mechanism remains largely unknown.

Phosphatase and tensin homolog deleted on chromosome 10 (PTEN) was first identified as a tumor suppressor and plays multiple regulatory roles in ovarian development, especially the control of oocyte activation [15, 16]. As a negative regulator of PI3K signaling, PTEN was also reported to be involved in the tumorigenesis of epithelial ovarian cancer and inversely correlated with ovarian cancer clinical outcome [15, 17, 18]. P27 is an inhibitor of cyclin-dependent kinase and functions as a tumor suppressor in multiple tumor types, including epithelial ovarian cancer, pituitary tumor, and endometrial carcinoma [19–21]. In the ovary, it is highly expressed in ovarian follicle pregranulosa cells and plays a vital role in controlling follicular development [22]. Interestingly, although PTEN and P27 are the key suppressors of tumorigenesis, deletion of either *Pten* or *p27* results in hardly any GCT formation in mice. It was reported that *Pten* and *p27* have a cooperative role in tumor suppression [23]. Whether PTEN and P27 cooperate in GCT formation is unknown.

In this study, we created a mouse model that shared similar symptoms with human GCT cases by inducible deletion of *Pten* and *p27* from GCs. Furthermore, we combined the multi-fluorescent *Rainbow* reporter mouse model with a GCT mouse model, and performed cell lineage tracing of mutant GCs which revealed a single mutant GC derived pattern of GCT tumorigenesis. RNA-Seq analysis of tumorigenesis showed that a molecular evolution occurred when mutant GCs transformed into GCTs, and the increase in innate immunity factors, such as *Cd47*, might determine the formation of GCTs. Therefore, inhibition of *Cd47* suppressed the GCT formation efficiently.

## Results

### Deleting *Pten* and *p27* from granulosa cells (GCs) to establish an inducible GC mutant mouse model

**Fig. 1:**
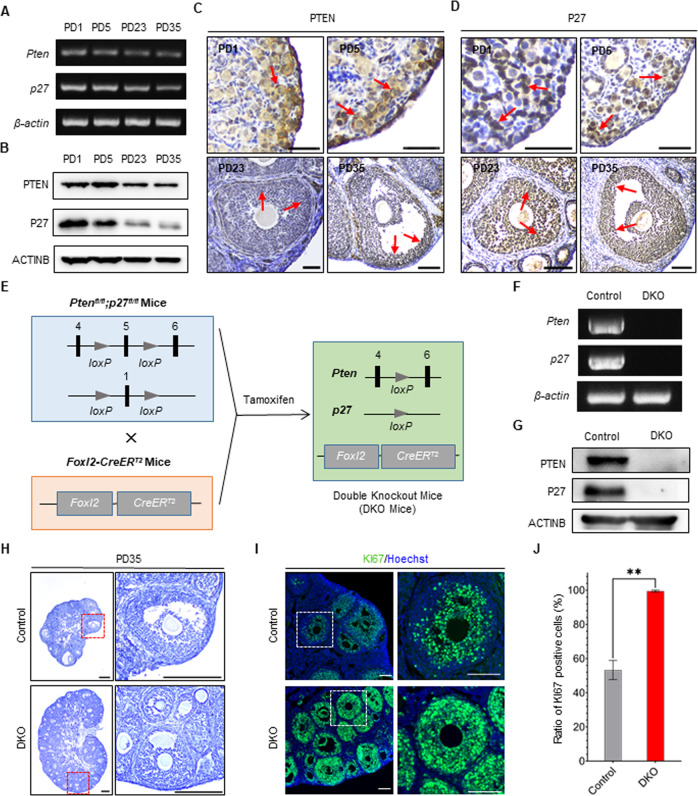
Deleting *Pten* and *p27* from granulosa cells (GCs) to establish an inducible GC mutant mouse model. **A** PCR results of *Pten* and *p27* transcripts in ovaries on different postnatal days (PDs) as indicated, showing a continuous transcription of both genes in the ovaries from newborn to adult. **B** Immunoblot of PTEN and P27 in ovaries on different PDs as indicated, showing a continuous expression of both proteins in the ovaries from newborn to adult. Immunohistochemistry of PTEN (**C**) and P27 (**D**) on PD1, PD5, PD23, and PD35, demonstrating a clear distribution of both proteins in (pre-)GCs from newborn to adult. Red arrows indicate (pre-)GCs. Scale bars: 100 μm. **E** Schematic diagram of the *Pten*^*fl/fl*^, *p27*^*fl/fl*^, and *Foxl2-CreER*^*T2*^ alleles and the conditional double knockout (DKO) mouse generated upon tamoxifen (Tam) administration on PD1, PD3, and PD5. **F** PCR results of *Pten* and *p27* in NoCre control and DKO GC genome DNA, revealing the efficient knockout of both genes in DKO GCs. **G** Immunoblot of PTEN and P27 in NoCre control and DKO GCs on PD23, revealing no expression of either protein in DKO GCs. **H** Representative hematoxylin-stained sections from NoCre control and DKO ovaries on PD35, showing numerous growing follicles developing in the ovaries of a DKO mouse one month after completion of Tam treatment, as described above. The boxed regions in the left panels are magnified in the right panels. Scale bar: 500 μm. **I** Immunofluorescence staining with KI67 (proliferation marker, green) and Hoechst (nuclear stain, blue) of ovaries from NoCre control and DKO mice on PD35, illustrating the excessive proliferative activity of the mutant GCs in the ovaries of DKO mice. Scale bar:  100 μm. **J** Statistical results of KI67-positive cells in follicles at the developmental stage from ovaries of NoCre control and DKO mice, confirming the conclusion stated in I. Data represent mean ± SD from three mice of each genetic background. **P < 0.01, unpaired *t* test.

The expression patterns of *Pten* and *p27*, which are well-identified tumor suppressor genes [15, 24], were first detected at different ages. At the transcriptional and translational levels, the PCR and western blot results showed that both *Pten*/PTEN and *p27*/P27 were expressed in the ovaries from newborn to adult (Fig. 1A, B and Supplementary Fig. S1A). Via immunostaining, we found that PTEN and P27 were mainly localized in the (pre-)GCs (Fig. 1C, D), which are FOXL2-positive cells in the ovaries. These results showed the continuous expression of PTEN and P27 in the ovarian GCs.

Next, we crossed the *Pten*^*fl/fl*^;*p27*^*fl/fl*^ mutant mice with a well-identified GC-specific inducible Cre mouse model (*Foxl2-CreER*^*T2*^) to generate a *Foxl2-CreER*^*T2*^;*Pten*^*fl/fl*^;*p27*^*fl/fl*^ mouse model (Fig. 1E). In this mouse model, the deletion of *Pten* and *p27* from GCs could be induced by tamoxifen (Tam) administration. The resulting mice are referred to as DKO mice, and the littermates of the NoCre;*Pten*^*fl/fl*^;*p27*^*fl/fl*^ females are referred to as control mice. To efficiently delete *Pten* and *p27* from (pre-)GCs, Tam was administrated to DKO females at postnatal day 1 (PD1), PD3, and PD5. PCR and western blot analysis showed that there was a complete loss of both *Pten*/PTEN and *p27*/P27 in the GCs of DKO females at PD23, demonstrating satisfactory deletion efficiency after neonatal Tam treatment (Fig. 1F, G). Therefore, the ovaries of DKO mice contained mutant GCs without the tumor suppressors PTEN and P27.

After Tam treatment, ovaries from DKO and control mice had similar morphologies. They had many primordial follicles at PD5, indicating that the neonatal deletion does not affect the formation of primordial follicles (Supplementary Fig. S1B). However, the ovaries of the DKO mice were much larger than those of the control mice from PD13 to PD35, and numerous growing follicles were seen in the ovaries of the DKO mice, showing a phenotype of follicle over-activation (Fig. 1H and Supplementary Fig. S1B). This is consistent with the previous findings on follicle over-activation in *p27*-knockout females [25]. Moreover, we found that the proliferating ratio of GCs in ovaries of DKO mice was significantly higher than that of GCs in ovaries of control mice at PD35, showing that deletion of *Pten* and *p27* led to excessive proliferation of mutant GCs in ovaries (Fig. 1I, J). Although a follicle over-activation phenotype was found in all mutant ovaries, we did not find any tumor-like structures in DKO females before adulthood.

### Granulosa cell tumors (GCTs) form in double knockout (DKO) females with relatively low incidence in a short time frame

**Fig. 2:**
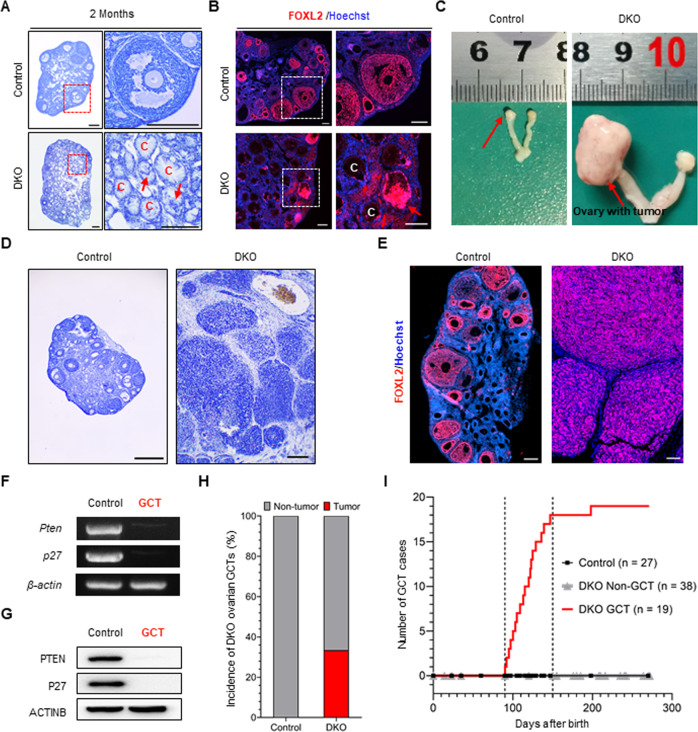
GCTs formed in DKO females with relatively low incidence in a short time frame. **A** Representative hematoxylin-stained sections of ovaries from NoCre control mice and DKO mice at two months after completion of Tam treatment, showing numerous cyst-like structures with cavities appearing in the ovaries of DKO mice. C indicates cyst-like structures. Red arrows indicate residual cells. The boxed regions in the left panels are magnified in the right panels. Scale bar: 500 μm. **B** Immunofluorescence staining of FOXL2 (GC marker, red) and Hoechst (nuclear stain, blue) in ovaries of NoCre control and DKO mice, showing residual cells were FOXL2 positive. The boxed regions in the left panels are magnified in the right panels. C indicates cyst-like structures. The red arrow indicates residual FOXL2-positive cells. Scale bar: 100 μm. **C** Representative images of genital ducts and ovaries dissected from NoCre control and DKO mice, showing an ovarian tumor that developed in a DKO mouse at three to four months. Red arrows indicate the normal ovary (left) and ovarian tumor (right). **D** Representative hematoxylin-stained sections of ovaries showing nest structures with clear outlines in a DKO ovarian tumor compared to a control ovary dissected from a NoCre control mouse of similar age. Scale bar: 500 μm. **E** Immunofluorescence staining of FOXL2 (red) and Hoechst (blue) in ovaries of NoCre control and DKO mice, showing that the tumor cells that developed in DKO females were FOXL2 positive. Scale bar: 100 μm. **F** PCR results showing that there was no *Pten* or *p27* in the GCT cell genome DNA from DKO mice compared to the NoCre control GC genome DNA. **G** Immunoblot results showing that PTEN and P27 were not expressed in GCT cells from DKO mice compared to NoCre control mice GCs. **H** Statistical results showing that the incidence of GCTs in DKO mouse ovaries was 33.3% (19/57) during the nine months of tracing. **I** Statistical results showing the number of GCT cases detected at the indicated days, illustrating that GCT tumorigenesis was concentrated in a short time frame from three to four months.

By 2 months of age, the ovaries of the DKO female mice contained many small cyst-like structures with cavities (Fig. 2A). Nuclear staining showed that most of the cysts contained only a few cells, and only a small proportion of the cysts were full of residual cells, which were rapidly dividing (Fig. 2A and Supplementary Fig. S2A). Immunostaining showed that these active cells were FOXL2 positive, indicating that the cysts were derived from the mutant GCs (Fig. 2B). Notably, although many abnormal cysts were found in the ovaries of the DKO females, there were no classical tumor-like structures in any of the ovaries at two months old.

From three months on, enlarged and solid ovaries were gradually detected in some of the DKO females. Dissection revealed that these ovaries usually appeared in hemorrhagic solid and/or cystic tumorous tissues and reached 0.8–1.2 cm in size, which was in sharp contrast to the normal ovaries (0.1–0.2 cm) found in the control females at the same age (Fig. 2C). To determine the characteristics of the enlarged ovaries, a histopathological analysis was performed and found that there were many distinguishable nest structures (size variation of 0.1–5 mm) with clear outlines in the enlarged ovaries (Fig. 2D). This was in contrast to the normal distribution of ovarian follicles found in the control ovaries. Immunofluorescent staining demonstrated that all cells in the ovarian nests were FOXL2 positive (Fig. 2E). The nuclei exhibited a highly uniform round/oval shape (Supplementary Fig. S2B), resembling the reported characteristics of human GCT cells [3, 26]. To identify whether the ovarian nest cells were derived from mutant GCs or other cells, we detected *Pten*/PTEN and *p27*/P27 in GCT cells from DKO females. None of the GCTs was found detectable for DNA or protein for *Pten*/PTEN or *p27*/P27, confirming that the GCTs were derived from mutant GCs in the ovaries of DKO females (Fig. 2F, G). Therefore, we concluded that the GCTs were formed in the DKO females three months after deletion.

Although a large number of mutant GCs were found to exist in the ovaries, only 35.6% ± 13.9% of the DKO females had GCTs during the nine months of the study (Fig. 2H), and in these mice, 84.2% bore unilateral tumors while others bore bilateral tumor (Supplementary Fig. S2C). The ovaries in the remaining DKO females were retarded in a state that resembled ovaries at two months old with many cyst-like structures (Supplementary Fig. S2D). Additionally, GCT incidence was found to be concentrated within a short time frame at the age of three to four months (Fig. 2I).

### GCT nests are derived from single mutant GCs in DKO females

**Fig. 3:**
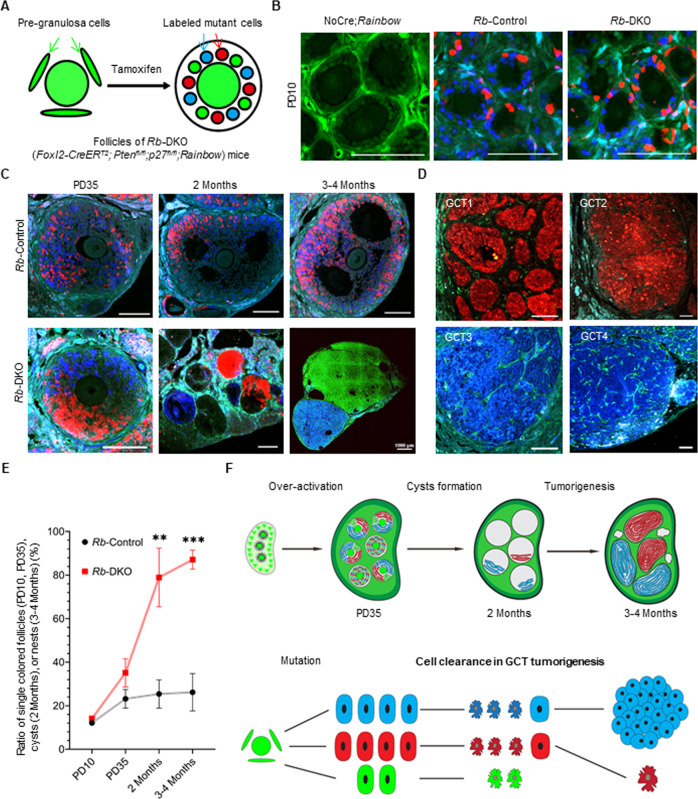
Granulosa cell tumor (GCT) nests are derived from single mutant granulosa cells (GCs) in DKO females. **A** Schematic diagram depicting the lineage tracing strategy using *Rb-*DKO (*Foxl2-CreER*^*T2*^*;Pten*^*fl/fl*^*;p27*^*fl/fl*^*;Rainbow*) mice. **B** Fluorescent detection in cross-sectioned ovaries from NoCre;*Rainbow*, *Rb*-Control, and *Rb-*DKO mice on PD10 with tamoxifen (Tam) induction on PD1, PD3, and PD5, showing specific and random labeling of GCs in ovaries of *Rb*-Control and *Rb-*DKO mice. **C** Fluorescent detection of randomly labeled GCs in ovaries from *Rb*-Control and *Rb*-DKO mice after Tam induction (PD1, PD3, and PD5) at indicated time points, revealing a clear single-cell origin pattern of GCT nests in *Rb*-DKO females. **D** Fluorescent detection in *Rb*-DKO mice three to four months after Tam treatment, showing single-color labeling of cysts in GCTs, which demonstrates that one mutant GC is enough to form a GCT in *Rb*-DKO females. **E** Statistical results of single-colored follicles, cysts, or nests determined at the indicated times in mice from each treatment group (*n* = 3 mice/group), confirming the conclusion stated in **C** and **D**. Data represent means ± SD, **P < 0.01, ***P < 0.001, two-way ANOVA. **F** Upper panel shows a schematic diagram illustrating the process of GCT formation: each GCT nest derives from a single mutant GC, and one mutant GC is enough to form a GCT. The lower panel shows a schematic diagram depicting a potential cleaning mechanism utilized during the development of mutant GCs into tumor cells. Most mutant GCs are removed by an internal cleaning mechanism, leaving a few mutant GCs to form GCTs by gaining unknown factors.

To explain the low incidence of GCT formation, we hypothesized that GCT formation requires the aggregation of multiple mutant GCs. To test this hypothesis, we introduced a multi-fluorescent *Rainbow* reporter into DKO mice to generate an in vivo mutant GC mouse model for tracing. In this genetic approach, Tam administration induces a *Foxl2-CreER*^*T2*^-mediated recombination at the *Rainbow* cassette, leading to a random change in expression from green EGFP to red RFP, orange OFP, or cyan CFP in the mutant GCs (Fig. 3A). This method allows us to trace the developmental fate of mutant GCs at the single-cell level and to identify the cellular origin of GCT nests by analyzing their fluorescent color in vivo. In this study, the *Foxl2-CreER*^*T2*^;*Pten*^*fl/fl*^;*p27*^*fl/fl*^*;Rainbow* females were termed *Rb*-DKO mice, and the *Foxl2-CreER*^*T2*^;*Rainbow* females were used as controls and were defined as *Rb*-Control mice.

To trace the development of mutant GCs, Tam was administered on PD1, PD3, and PD5 to the *Rb*-DKO and *Rb*-Control females. On PD10, in most of the ovarian follicles of the *Rb*-DKO and *Rb*-Control females, GCs were labeled with CFP or RFP; however, this was not observed in the ovaries of negative control NoCre; *Rainbow* females (Fig. 3B). These fluorescently labeled GCs were localized in the follicles with a mixed-color pattern, showing a random labeling after Tam-induced recombination. In terms of follicular development, we found that on PD35, the randomly distributed model of labeled GCs continued in follicles from the secondary to antral stages in both *Rb*-DKO and *Rb*-Control females (Fig. 3C). In *Rb-*DKO mice at two months of age, the majority of cysts (78.9%) that contained cells were labeled with a single color in the mutant ovaries (Fig. 3C). This result strongly implies that the surviving cells in cysts are derived from single mutant cells.

Regarding GCT development, GCTs were detected with enlarged ovaries in around 30% of *Rb*-DKO females, and the incidence was similar to that in DKO females, suggesting that the *Rb* cassette did not affect GCT formation. In line with the single-color labeling of cysts in ovaries at two months, almost all of the GCT nests were composed of single-color labeled cells (Fig. 3C, D). Statistical analysis confirmed that the majority of the GCT nests were composed of single-color labeled cells, which was in sharp contrast to the follicles in *Rb*-Control females that had mixed-color labeled cells (Fig. 3E). This result suggests that each GCT nest is derived from a single mutant GC, and that one mutant GC is enough to form a GCT. Furthermore, our findings imply that there is a cleaning mechanism during the development of mutant GCs in the ovaries of DKO mice: an internal clean-up mechanism removes most of mutant GCs, leaving only a few mutant GCs to survive and form single cell derived GCTs by gaining unknown factors (Fig. 3F).

### The pathways of apoptosis and PGCCs formation contribute to single cell derivation of GCT

**Fig. 4:**
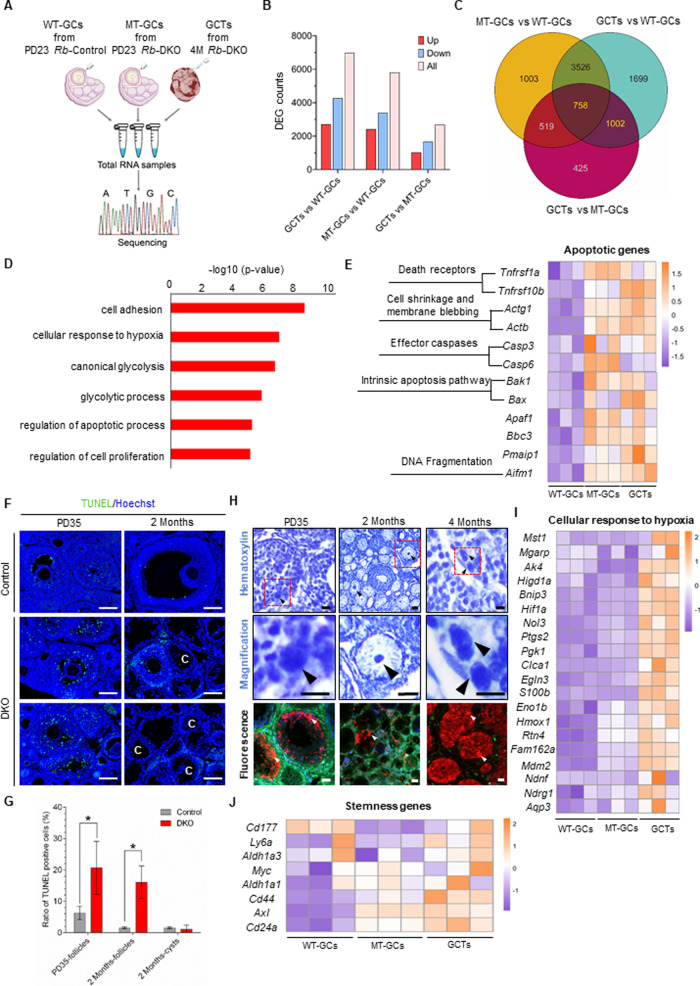
Apoptosis and PGCCs formation contribute to single cell derivation of GCT. **A** Schematic diagram depicting the process used to conduct RNA-Seq analysis on cells from the indicated types of ovaries (*n* = 3 for each group). **B** Bar graph of the number of differentially expressed genes (DEGs) found when WT-GCs, MT-GCs, and GCTs were compared, showing that GCTs and MT-GCs shared the lowest number of DEGs among the three compared groups. **C** Venn diagram showing the number of DEGs in the MT-GCs vs. WT-GCs, GCTs vs. WT-GCs, and GCTs vs. MT-GCs comparisons, revealing that 1,760 DEGs were up/downregulated in GCTs compared to those in WT-GCs and MT-GCs. **D** Significantly enriched pathways of the up-regulated differentially expressed genes (DEGs) between GCTs and the other two groups. Data were analyzed by DAVID Bioinformatics Resources 6.8 for Gene Ontology (GO) enrichment analysis of DEGs. GO terms with corrected *P*-values that lower than 0.05 were considered significantly enriched by DEGs. **E** Heat map of apoptotic genes in WT-GCs, MT-GCs and GCTs groups, showing upregulation of apoptosis related genes during tumorigenesis. **F** TUNEL detection in ovaries of No-Cre control and DKO mice, demonstrating the upregulation of apoptosis signals during cyst formation. Representative images are shown. Scale bar: 100 μm. **G** Statistical results of **F**. Data represent mean ± SD. *P < 0.05, unpaired *t* test. **H** Hematoxylin staining and fluorescent detection of ovaries at different developmental stage of *Rb*-DKO ovaries, showing PGCCs (arrowheads) formation was involved in the tumor formation. Boxed regions in upper panels are magnified in the lower panels. Scale bar: 10 μm. **I** Heat map of genes responses to hypoxia among WT-GCs, MT-GCs and GCTs, indicating a high level of hypoxia response in GCTs. **J** Heat map of stemness genes among WT-GCs, MT-GCs and GCTs, illustrating cancer stem cells were involved in tumor formation and progression.

To reveal key cellular components and pathways responsible for the single cell derivation of GCT, we collected mutant GCs from ovaries of PD23 DKO mice (MT-GCs) and GCT cells from four-month-old DKO females with tumors (GCTs). GCs from ovaries of PD23 control mice (WT-GCs) were used as normal controls. RNA-Seq was performed to analyze the transcriptomic gene expression profiles of those cells (Fig. 4A). Analysis of differentially expressed genes (DEGs) identified 5,806 DEGs between WT-GCs and MT-GCs (Fig. 4B). These data suggested that loss of *Pten* and *p27* caused abnormal expression of a large number of genes; however, these changes did not drive the GCs to transform into GCTs. In contrast to the DEG results of the WT-GCs and MT-GCs, there were 2,686 DEGs identified between the MT-GCs and GCTs, and 6,985 DEGs identified between the WT-GCs and GCTs. Venn diagram analysis showed that 758 DEGs were up/downregulated in all comparisons, and there were differences in 1,002 DEGs when MT-GCs were compared with GCTs (Fig. 4C). The expression patterns of these collective 1,760 DEGs (758 plus 1,002) suggested that these genes are candidates for causing GCT transformation. Therefore, we focused on these genes when performing further analysis.

Among these 1,760 DEGs, unsupervised analysis between GCTs and the other two groups showed that 873 genes were downregulated and 771 genes were upregulated (Supplementary Fig. S3). GO analysis of the upregulated DEGs showed that genes associated with terms including “cell adhesion”, “cellular response to hypoxia”, “regulation of apoptotic process” and “regulation of cell proliferation”, were significantly enriched (Fig. 4D). Given the drastic loss of GCs during cyst formation, we paid attention to the term “regulation of apoptotic process”, as shown in Fig. 4E, apoptotic genes such as *Casp3, Bak1* and *Bax* were upregulated in both MT-GCs and GCTs. We then detected the apoptotic cells during cyst formation via TUNEL staining, a method widely used to detect apoptotic nuclei in situ [27]. As shown in Fig. 4F, G, the percentage of TUNEL positive cells in follicles was significantly higher in DKOs than their comparative controls at PD35 (20.63 ± 8.44% v.s. 6.28 ± 2.14%) and 2 Months (16.03 ± 5.23% v.s. 1.51 ± 0.27%), whereas the TUNEL positive apoptotic cells in cysts were not apparent at 2 months (1.12 ± 1.23%) in DKO ovary, indicating that most of the mutant GCs underwent apoptosis, only a small part of cells survived to form GCTs.

Notably, the polyploid giant cancer cells (PGCCs), multinucleated or mononucleated, were extensively exist in GCTs since PD35 via looking into sections with hematoxylin staining or directly from tumor sections of *Rb-*DKO mice (Fig. 4H). The formation of PGCC was reported to be induced under stress and acted as tumor initiating cells that express cancer stem cell markers [28–31]. Interestingly, we observed that the genes related to hypoxia such as *Hif1a, Egln3, ptgs2* and *pgk1* and stemness markers including *CD44, ly6a, Axl* was enriched in GCTs compared to normal GCs and mutant GCs (see Fig. 4I, J). Together, these data indicating that a small part of cells survive hypoxia stress and form PGCCs, which fuels tumor formation and progression.

### Immune evasion with up-expressions of CD24 and CD47 is involved in GCT formation

**Fig. 5:**
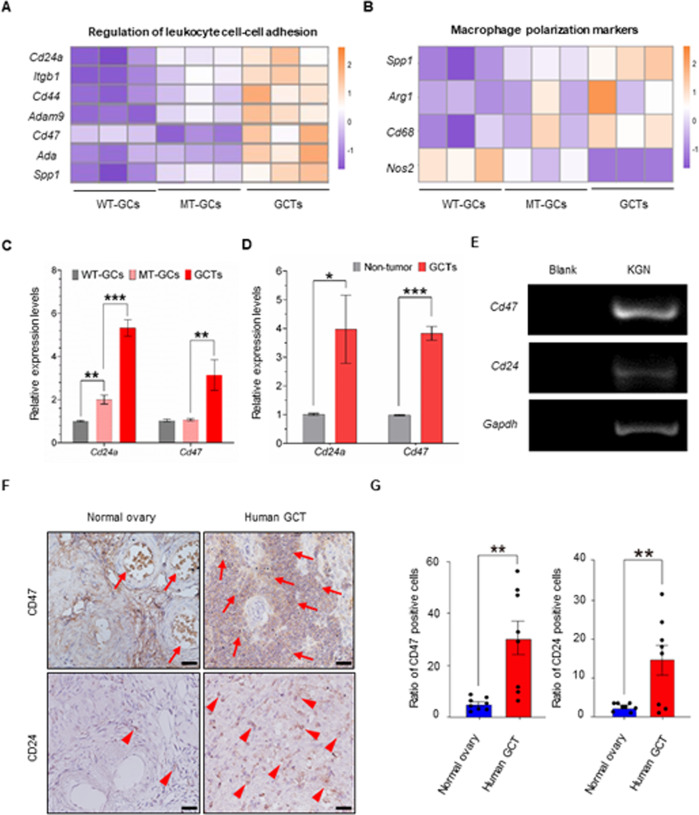
Immune evasion is involved in GCT formation. **A** Heat map revealing increased expressions of *Cd24a* and *Cd47* in GCT cells. **B** Heat map of regulation of macrophage polarization markers associated genes in different groups, indicating the imbalance of M1/M2 macrophages in the GCTs. **C** q-PCR analysis of *Cd24a* and *Cd47* expression in indicated groups, confirming the transcriptomic result of high *Cd24a* and *Cd47* mRNA levels in GCT cells. Data indicate means ± SD; **P < 0.01; ***P < 0.001, two-way ANOVA. **D** Relative *Cd24a* and *Cd47* mRNA expression levels in the non-tumor DKO ovaries and GCTs, showing higher expression levels of both *Cd24a* and *Cd47* in GCTs than that in the non-tumor DKO ovaries. Data indicate means ± SD, *P < 0.05; ***p < 0.001, unpaired *t* test. **E** PCR results of *CD47* and *CD24* transcripts in the KGN cell compared to the control, showing the expression of *CD47* and *CD24* in KGN cell line. Blank: negative control. **F** Immunochemistry staining of CD47 (arrows) and CD24 (arrowheads) in human normal ovaries and GCTs, showing more CD47 and CD24 positive cells in the GCT tissues. Scale bar: 100 μm. **G** Statistic results of **F**. Data represent mean ± SD. **P < 0.01, unpaired *t* test.

To investigate the underlying molecular mechanisms of GCTs formation, we reanalyzed the “cell adhesion” term, which was the most significant enriched term according to GO analysis by comparing GCTs to other two groups (Fig. 4D). Among hub genes in this term, the “do not eat me” genes *CD24a*, *Cd47* and the macrophage associated genes *CD44*, *Itgb1*, *Adam9* and *Spp1* were specifically enriched, these genes were identified as “leukocyte cell-cell adhesion”, a sub GO type of “cell adhesion” (Fig. 5A, Supplementary Fig. S4A). The significant upregulation of *Cd24a* and *Cd47* in GCTs were of particular interest due to their role in evading phagocytic elimination by macrophages that proved in extensive other tumors [32–34]. Interestingly, we further analyzed the subpopulation of macrophages in GCTs and observed that the M2-like macrophage regulators such as *Spp1* (promotes macrophage M2 polarization), *Arg1*, were highly expressed, whereas *Nos2*, the marker of M1-like polarized macrophages that exhibited high level of phagocytic activity, was down-regulated in GCTs, this imbalance of M1/M2 may induce inhibited phagocytosis (see Fig. 5B). Together, these data indicate that CD24a/CD47 serve as important regulators in escaping macrophage mediated phagocytosis during the transformation of mutant GCs into GCTs.

The higher expression of *Cd24a* and *Cd47* in GCTs than MT-GCs and WT-GCs observed from RNA-Seq was further confirmed by q-PCR (Fig. 5C, D). To clarify the tumor expression of *Cd24* and *Cd47* in human GCTs, we performed q-PCR on KGN cell line, which originated from a Stage III granulosa cell carcinoma [35]. As shown in Fig. 5E, both *Cd24* and *Cd47* were detected in KGN cells, suggesting the *Cd24* and *Cd47* were expressed in tumor cells of human GCTs. To assess whether the dysregulation of the two proteins found in our mouse model exists in human GCTs, we compared our DEGs with those from two datasets associated with human granulosa cell tumor development [36] or *FOXL2* mutation [8]. As shown in the Supplementary Fig. S4B, comparison of our DEGs with those between human progressive GCT (1 case) and normal ovaries or between *Foxl2*^*mut*^ and *Foxl2*^*wt*^ GCTs (6 pairs) identified fewer common genes, 188 and 130 co-upregulated genes, respectively. Unfortunately, we did not find a significant increase of *Cd47* in the above human GCT datasets, probably due to the extensive tumor variation and limited subject numbers analyzed. To solve this issue, we collected human GCTs and comparable control ovaries (8 pairs, details in supplementary clinical information), and performed IHC (Immunohistochemistry) of CD47 and CD24 on paraffin slides of these human subjects. As shown in Fig. 5F, G, both proteins were rarely expressed in granulosa cells, but increased in most GCTs (5/8), indicating the upregulation of these proteins exist in at least some human GCTs.

### In vivo inhibition of *Cd47* results in a reduction of tumor growth

**Fig. 6:**
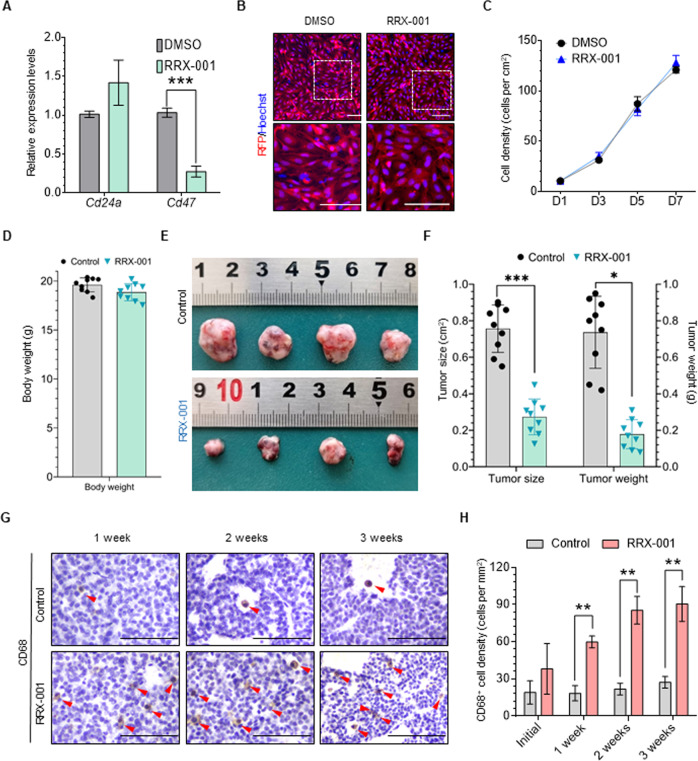
In vivo inhibition of *Cd47* results in a reduction of tumor growth. **A** Relative mRNA expression levels of *Cd24a* and *Cd47* in in vitro cultured GCT cells treated with RRX-001, with DMSO-treated GCT cells serving as the control. The result shows that RRX-001 treatment efficiently suppresses *Cd47* expression in GCT cells. Data indicate means ± SD, ***p < 0.001, unpaired *t* test. **B** Representative morphologies of in vitro cultured GCT cells in DMSO and after RRX-001 treatment, showing that the proliferation of GCT cells was not affected by RRX-001 treatment. The boxed regions in the upper panels are magnified in the lower panels. Scale bar: 100 μm. **C** The statistical results of cell proliferation during 7 days culture, showing that the proliferation of GCT cells was not affected by RRX-001 treatment. Data represent mean ± SD. **D** Body weights of vehicle control and RRX-001-treated mice, showing comparable body weights of the RRX-001-treated and control groups. RRX-001 was administrated every two days for three consecutive weeks (*n* = 9). Data indicate means ± SD. **E** Representative images of tumors dissected from mice treated with vehicle and RRX-001, illustrating that the GCTs in the RRX-001-treated group were significantly smaller than those in the vehicle control group. **F** Statistical results of the size and weight of tumors from the vehicle control and RRX-001-treated groups, demonstrating that RRX-001 treatment severely inhibits GCT growth in vivo. **G** Immunohistochemical staining of CD68 (macrophage marker) in GCT sections from the vehicle control and RRX-001-treated groups, revealing a greater number of invasive CD68 + macrophages in the GCTs from the RRX-001-treated group than from the vehicle control group. Red arrowheads indicate CD68 + macrophages. Scale bar: 100 μm. **H** Cell density of CD68 + macrophages in indicated groups, confirming the conclusion stated in **G**. Data indicate means ± SD, **p  <  0.01, unpaired *t* test.

Next, we used a well-identified *Cd47* inhibitor (RRX-001) to test whether inhibiting the immune evasion-related factors could suppress GCT growth. In the cultured GCT cells, we found that RRX-001 treatment efficiently suppressed *Cd47* expression (Fig. 6A, B and Supplementary Fig. S5A, B). However, cell proliferation was not affected, showing that RRX-001 could not directly suppress GCT cell proliferation (Fig. 6C). To further explore whether blocking CD47 enhances the therapeutic antitumor response in vivo, we subcutaneously transplanted GCT cells (5 × 10^6^ cells per mouse) isolated from four-month-old *Rb*-DKO females to severe combined immunodeficiency (SCID) mice (T cell-, NK cell-, and B cell-deficient) to establish a heterotopic GCT model. As expected, the transplanted cells formed tumors (0.55–0.90 cm^2^) four weeks after surgery, showing the successful establishment of the heterotopic GCT model. Next, one week after transplantation, we treated the mice with or without RRX-001 (10 mg/kg BW, every other day, i.p.) for three weeks. After treatment, the body weights of the RRX-001-treated and control mice were similar, showing that the RRX-001 treatment had few side effects on the animals (Fig. 6D). However, both tumor size and weight were significantly lower in the RRX-001-treated group than in the control group (size: 0.82 ± 0.07 cm^2^ in control vs. 0.29 ± 0.13 cm^2^ in RRX-001 treated; tumor weight: 0.61 ± 0.24 g in control vs. 0.15 ± 0.08 g in RRX-001 treated) (Fig. 6E, F). This result clearly shows that RRX-001 treatment inhibits GCT cell growth in vivo. Consistently, syngeneic transplantation of GCT cells into C57BL/6 mice showed similar responses to RRX-001 treatment (Supplementary Fig. S5C, D).

Previous studies had reported that RRX-001 exhibits potent anti-cancer activity on tumors dependent on the presence of tumor-associated macrophages in tumor tissue [37, 38], so we assessed the macrophages indicated by CD68 in GCTs treated before and after RRX-001. As shown in Fig. 6G, H and Supplementary Fig. S5E, we found a greater number of invasive CD68 + macrophages in the tumors of the RRX-001-treated group than in the control group as early as 1 week after RRX-001 treatment (2 weeks after transplantation), showing that suppressing *Cd47* in GCT cells dramatically increases macrophage invasion. To confirm the RNA-Seq data in Fig. 5B, we performed IHC staining of the CD206 and iNOS, the markers of M2 and M1 macrophage, on tumor sections with/without RRX-001 treatment, respectively. As shown in Supplementary Fig. S5F and G, RRX-001 treatment significantly increased the expression of iNOS, suggesting that RRX-001 skews macrophage differentiation and stimulate phagocytosis in GCTs. These data in consistent with previous studies [32, 33]. The above results demonstrate that RRX-001 treatment is an efficient strategy for suppressing GCT formation in mice by inhibiting *Cd47* expression.

## Discussion

Although patients with early-stage GCTs have a very good prognosis, those with recurrent chemoresistant, progressive non-responding tumors or high surgical risks are still in urgent need of novel therapies. Unlike well-examined epithelial ovarian cancer, little is known about the mechanisms underlying the formation and development of GCTs. In this study, we generated a GCT model by conditionally knocking out *Pten* and *p27* in FOXL2 + GCs. Consistent with the clinical features of human GCTs, the incidence of GCT formation from mutant GCs in our DKO mouse model was low (approximately 30%) and most of the tumors (84.2%) were found unilateral. It is in accordance with the clinical features of human GCTs, with unilateral tumor occurs in approximately 90% patients and bilateral tumor appears in just less than 10% patients [5, 26]. Genetic intratumor heterogeneity as an inevitable consequence of the dynamic tumor evolution likely responsible for this tumor variation [39, 40]. Tumor cells can adapt plastically to different environments such as hypoxia or the presence of immune systems [41–44]. Our further studies showed that GCTs are derived from single mutant GCs that escape immune surveillance by expressing higher levels of *Cd47* and *Cd24a*, while blocking CD47 in mice bearing GCTs efficiently decreases tumor size.

Through cell linage tracing using fluorescent labeling, we found that single-colored clones were distributed throughout the whole tumor, raising the possibility of the existence of specific “stemlike” cells that act as tumor-initiating cells. In this study, we observed that GCTs harbor PGCCs, which had been reported as tumor initiating cells that express cancer stem cell markers [28–31]. These cells could generate regular-sized cancer cells quickly through asymmetric cell division, a depolyploidization way named neosis [45]. Indeed, our RNA-Seq data showed that there is a higher expression of stemness markers including *Cd44*, *ly6a*, *Axl* in MT-GCs and GCTs when compared with WT-GCs. The enrichment of PGCCs and cancer stem cell markers in GCTs indicate that the tumors might be generated from single cancer stem cells, which consistent with the single cell derivation of tumor formation observed from our lineage tracing result. Nevertheless, a more comprehensive understanding of the molecular mechanisms underlying polyploidization in GCTs and how PGCC involve in tumor progression need to be further investigated.

Macrophages are the most abundant immune cells in the ovaries, and they have important functions in the innate immune system. CD47, with its ligand signal regulatory protein a (SIRPa), mainly functions as an anti-phagocytic or “do not eat me” signal. This enables CD47-expressing cells to evade phagocytic elimination by macrophages and other phagocytes. The overexpression of CD47 has been identified in many tumors, and its level positively correlates with tumor evasion and metastasis [46–49]. Hence, there is considerable interest in targeting CD47 and its associated signals as a therapeutic strategy; for example, blocking CD47 was found to significantly inhibit tumor growth and metastasis [50]. Consistent with these findings, in our study, higher CD47 expression was observed in mutant GCTs than in GCs harboring similar mutations, indicating its potential role in blocking phagocytosis and, thus, evading immune surveillance. Furthermore, we have shown that treating immunocompetent mice that have GCTs with RRX-001, a downregulator of the CD47-SIRPa checkpoint pathway, induces efflux of CD68 + macrophages and efficiently dampens tumor growth, indicating that CD47-SIRPa could be a potential therapeutic target for GCT treatment. RRX-001 treatment did not affect the cell proliferation of GCTs cultured in vitro, but significantly dampened tumor growth in vivo, indicating that the anti-cancer activity of RRX-001 requires the exist of tumor-associated macrophages, which consistent with previous studies [37, 38]. The treatment of RRX-001 skews macrophage differentiation to M1 macrophage and stimulates phagocytosis in GCTs in vivo. In addition to CD47, we found that CD24 (another anti-phagocytic factor) was highly expressed in GCTs. Although CD24 inhibits phagocytosis as potently as CD47, it functions by inhibiting its own signals via binding to sialic acid-binding Ig-like lectin 10 (Siglec-10) [34]. Previous studies have reported that CD24 and CD47 are independent of each other and that simultaneous inhibition of both enhances phagocytosis in a synergistic manner [51]. As such, it would be interesting to explore whether the inhibition of CD24 and CD47 have different antitumor effects on GCTs.

In summary, this study has thoroughly elucidated the developmental process of GCT formation and shown that “do not eat me” genes act as underlying molecular determinants for GCT formation. The findings provide critical information for the development of new therapeutic strategies for the treatment of GCTs.

## Materials and methods

### Animals

C57BL/6 mice were purchased from the Laboratory Animal Center of the Institute of Genetics (Beijing, China). Previously described *Foxl2-CreER*^*T2*^ (*FOC*) mice were crossed with *Pten*^*fl/fl*^ and *P27*^*fl/fl*^ mice to obtain *Foxl2-CreER*^*T2*^;*Pten*^*fl/fl*^;*p27*^*fl/fl*^ mice. *Pten* and *p27* genes could be inducibly deleted in the GCs following tamoxifen administration. Thus, the resulting *Foxl2-CreER*^*T2*^;*Pten*^*fl/fl*^;*p27*^*fl/fl*^ mice are referred to as DKO mice. *Rainbow* mice [52] were crossed with DKO mice to generate *FOC;Pten*^*fl/fl*^*;P27*^*fl/fl*^*;Rainbow* (*Rb-*DKO) mice. NoCre;*Pten*^*fl/fl*^;*p27*^*fl/fl*^ and *Foxl2-CreER*^*T2*^;*Rainbow* females were used as control mice. *Foxl2-CreER*^*T2*^, *Pten*^*fl/fl*^, *p27*^*fl/fl*^, and *Rainbow* mice were generously gifted by Dr. Kui Liu.

All mouse strains were bred at C57BL/6 background. All mice were housed in standard SPF conditions under 16/8-h light/dark cycles at 26 °C with access to chow and water at libitum. The animal experiments conformed to the guidelines and regulatory standards of the Institutional Animal Care and Use Committee of China Agricultural University, No. AW52301202-3-1.

### Cell lines and patient samples

Human derived GCT cell lines KGN was kindly gifted by Dr. Chao Wang. KGN was cultured in DMEM/F-12 medium supplemented with 10% fetal bovine serum (FBS), penicillin (100 U/ml) and streptomycin (100 mg/ml), maintained at 37 °C in 5 % CO_2_.

All tissue samples used for this study were obtained with written informed consent from all participants and strictly followed guidelines and regulatory standards of the Medical Ethics Committee of The Third Affiliated Hospital of Guangzhou medical university, Medical Research (No. 20210723). Clinical information for the patient sample included in this paper are given in supplementary materials.

### Tamoxifen (Tam) administration

Neonatal (PD1, PD3, and PD5) mice were given three doses of tamoxifen (20 mg/kg body weight) every other day. Newborn pups were injected through stomach. Clinical observation and palpation three times weekly were conducted to monitor signs of tumor presenting before palpable tumors emerged. Once palpable tumors are present, daily clinical observation and palpation were carried out until euthanasia and sample collection.

### Western blot analysis

For the expression analysis of PTEN and P27 proteins, ovaries of PD1, PD5, PD23, and PD35 C57BL/6 mice were isolated and collected. For the deletion analysis of *Pten* and *p27* genes, granulosa cells of DKO females at PD23 and GCT cells of DKO females at 3-4 months were isolated and collected, respectively. For expression analysis of CD47 protein after RRX-001 treatment, tumor cells of 4 months GCT were collected and primarily cultured. Primary cells were collected following RRX-001 and DMSO treatment. Total proteins were extracted by using WIP lysis solution (1:10000) and Cell lysis solution (BioChip, 110000) according to the manufacturer’s protocol. Western blot experiments were conducted following standard procedure. The results were captured by using the SuperSignal chemiluminescent detection system (Thermo Scientific, 32109).

### Gene expression analysis

To detect the expression of *Pten* and *p27* genes in physiological conditions, ovaries of PD1, PD5, PD23, and PD35 C57BL/6 mice were isolated and collected. To detect the deletion state of *Pten* and *p27* genes, granulosa cells of DKO females at PD23 and GCT cells of DKO females at 3-4 months were isolated and collected, respectively. Total RNA was isolated by TRIZOL Reagent (Thermo-Ambion, 15596018) according to the manufacturer’s instruction. cDNA samples were obtained by using PrimeScript™RT reagent Kit with gDNA Eraser (TAKARA, RR047Q) following manufacturer’s instruction. Applied Biosystems 7500 Real-Time q-PCR System (Applied Biosystems, 4472908) was used to analyze the expression levels of individual genes.

### Histological analysis

For morphological analysis, ovaries and tumor tissues were fixed in 4% paraformaldehyde (Santa Cruz, 30525-89-4) for 8–24 hours (according to their sizes) at 4 °C, embedded in paraffin, and sectioned serially at 5–8 μm. Sections were stained with hematoxylin (Santa Cruz, sc-24973A) for further histological analysis.

### Immunofluorescence and immunohistochemistry staining

For immunofluorescence detection, ovary and tumor sections were deparaffinized and rehydrated. Antigen retrieval was conducted by high-temperature (95–98 °C) in 0.01% sodium citrate buffer (pH 6.0) for 16 min. 10% donkey serum (Jackson ImmunoResearch, 017-000-121) in PBS was used to block the sections for 60 min at room temperature. Primary antibody incubation was performed at 4 °C overnight. Sections were then incubated by secondary antibodies (1:200, Life Technologies) of corresponding species for 60 min at room temperature. Hoechst 33342 (HOE) was used as a nuclear counterstain. Sections were sealed with an anti-fade fluorescence mounting medium. For immunohistochemistry analysis, ovary sections were handled by using rabbit streptavidin-biotin method detection system (ZSGB-Bio, SP-9001) according to the manufacturer’s protocol.

### Primary cell culture and administration

For primary ovarian GCT cell culture, tumor tissues were digested into single cells in 10 mg/mL collagenase dissolved in PBS. Isolated cells were washed twice, centrifuged at 2000 *g* for 5 min and resuspended in DMEM/F-12 medium supplemented with 10% charcoal-stripped fetal bovine serum (Biological Industries, 04-201-1B), penicillin (100 U/ml), and streptomycin (100 mg/ml). GCT cells were collected and cultured in the same complete medium as the primary granulosa cells were seeded in. 5 μM RRX-001 dissolved in DMSO was added in the medium 24 hours post seeding. Cellular total RNA was collected 24 hours later for further q-PCR analysis. For cell counting, GCT cells were digested by 0.25% Trypsin-EDTA (Gibco, 25200072) for 1–3 min at 37 °C. Cell numbers of each group were measured by hemocytometer.

### RNA sequencing

Total RNA samples of ovaries, GCT tissues, and GCT cells were extracted by TRIZOL Reagent (Thermo-Ambion, 15596018). Degradation and contamination of RNA samples were monitored on 1.5% agarose gel. RNA purity was analyzed by using the NanoPhotometer ® spectrophotometer (IMPLEN, CA, USA). RNA integrity was detected by using the RNA Nano 6000 Assay Kit of the Bioanalyzer 2100 system (Agilent Technologies, CA, USA). RNA sample preparations were conducted by using a total amount of 1 µg RNA per sample as input material. NEBNext® UltraTM RNA Library Prep Kit for Illumina® (NEB, USA) was used to generate sequencing libraries according to manufacturer’s recommendations and index codes were added to attribute sequences to each sample. The cBot Cluster Generation System using TruSeq PE Cluster Kit v3-cBot-HS (Illumia) was utilized to perform the clustering of index-coded samples. After cluster generation, the Illumina Novaseq platform was used to sequence the library preparations and 150 bp paired-end reads were generated. The data were deposited in the NCBI Gene Expression Omnibus and are accessible through GEO Series accession number GSE220947.

Differential expression analysis was conducted by using the DESeq2 R package (1.16.1). The resulting P-values were adjusted for controlling the false discovery rate by using Benjamini and Hochberg’s approach.

### GCT cell transplantation and RRX-001 treatment

For GCT cell transplantation, cultured cells were digested into single cells by 0.25% Trypsin-EDTA when confluence about 70–80% was reached. Single cells were centrifuged at 2000 *g* for 5 min and resuspended into Matrigel matrix (Corning, 356243) at 4 °C to a final concentration of 5 × 10^7^ cells/ml. 100 μl cell suspension (5 × 10^6^ cells) was subcutaneous injected into the right interscapular region of each mouse. 1 week after transplantation, 10 mg/kg body weight RRX-001 was intraperitoneal injected into a part of recipient SCID females. The same amount of solvent (5% DMSO: 95% sterile water) was intraperitoneal injected into the other part of recipient SCID females which was referred as control. The development of transplanted GCT cells was checked three times a week and the size of tumors was measured with calipers. The recipient females were monitored for up to 8 weeks post transplantation.

### Statistics

All experiments were repeated at least three times. Sample organism participants were randomly allocated into experimental groups. Investigators were blinded to group allocation during data collection and analysis. Data are presented as the mean ± standard deviation (SD) of each result and no data were excluded. Data were calculated by Student’s *t*-test or two-way ANOVA, and were regarded statistically significant at *P* < 0.05. *P* is suggested as follows: * (*P* < 0.05), ** (*P* < 0.01), *** (*P* < 0.001) and n.s. (not significant, *P* ≥ 0.05). Statistics and charts were gained by using Prism 5 (GraphPad Software, La Jolla).

## Supplementary information

Supplementary figures and legends

Supplementary clinical information

Original westernblot images

Agreement on author changes

checklist

cdd-author-contribution-form

## Data Availability

All data are available in the main text or the supplementary materials.
