# Supplementary material for: Lineage tracing of mutant granulosa cells reveals in vivo protective mechanisms that prevent granulosa cell tumorigenesis
Source: Cell Death Differ. 2023 Feb 23;30(5):1235–46. doi: 10.1038/s41418-023-01132-1 (PMC10154338; doi:10.1038/s41418-023-01132-1)
Supplement: Supplementary file 1 — Supplementary figures and legends [file 41418_2023_1132_MOESM1_ESM.docx]

**Supplementary information**

**Lineage tracing of mutant granulosa cells reveals *in vivo* protective mechanisms that prevent granulosa cell tumorigenesis**


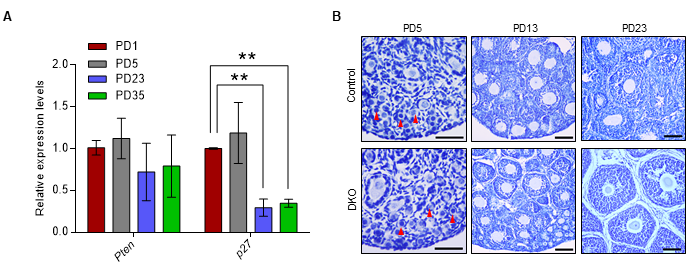


**Supplementary Fig. S1.** (related to Figure 1) **A,** Relative expression levels of *pten*, *p27* at different postnatal days by q-PCR detection. Confirming the results in Figure 1A. Data represent mean ± SD. **P < 0.01, unpaired *t* test. **B,** Representative hematoxylin-stained section from control and DKO ovaries, assessed at PD5, PD13 and PD23, respectively. These results show that neonatal deletion of *Pten* and *p27* does not affect the formation of primordial follicles at PD5, but can lead to a phenotype of follicle over-activation in DKO females at PD13 and PD23. Red arrowheads indicate primordial follicles. Scale bar: 100 μm.

**
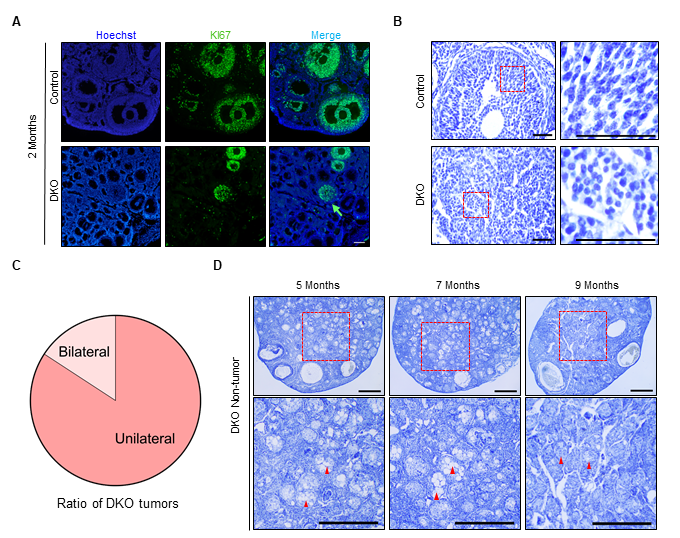
**

**Supplementary Fig. S2.** (related to Figure 2) **A**, Immunofluorescence staining for KI67 (proliferation marker, green) and Hoechst (nuclear stain, blue) in control and DKO ovaries, showing that the residual cells in DKO ovaries are still fast dividing. Green arrow indicates residual cells. Scale bar: 100 μm. **B**, Representative hematoxylin-stained section from NoCre control and DKO ovaries, revealing that the cellular nuclei exhibit highly uniform round/oval shape in nest structures of the DKO ovarian tumors. Boxed regions in left panels are magnified in the right panel of each pair. Scale bar: 50 μm. **C**, Pie graph of the ratio of the DKO tumors, showing most of the tumors were found unilateral. **D**, Representative hematoxylin-stained section from DKO ovaries at indicated time after completion of Tamoxifen treatment, illustrating that the non-tumor DKO ovaries are kept in a retarded state similar as the ovaries at 2 months old with many cyst-like structures from 5 months through to 9 months of age. Boxed regions in upper panels are magnified in the lower panel of each pair. Red arrowheads indicate cyst-like structure. Scale bar: 500 μm.

**
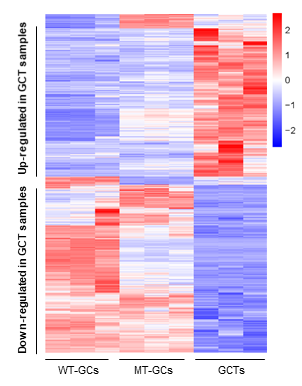
**

**Supplementary Fig. S3.** (related to Figure 4) Heat map showing transcripts (log-transformed TPMs) of the DEGs in representative ovaries/tumors from each indicated genotype, illustrating that there were 873 downregulated and 771 upregulated DEGs identified as candidates responsible for GCT tumorigenesis.

**
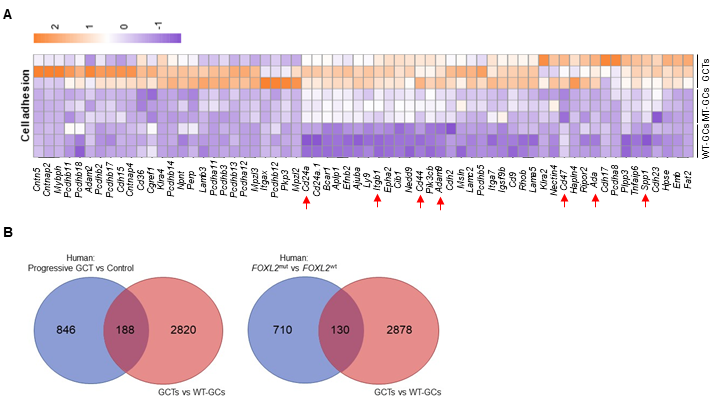
**

**Supplementary Fig. S4.** (related to Figure 5) **A,** Heat map of cell adhesion associated genes in different groups, showing the “cell adhesion” associated genes were upregulated in GCTs. **B,** Venn diagram showing the comparison of DEGs between GCTs and WT-GCs with those between the human GCT and normal ovaries or between the *FOXL2^mut^* and *FOXL2^wt^* GCTs.

**
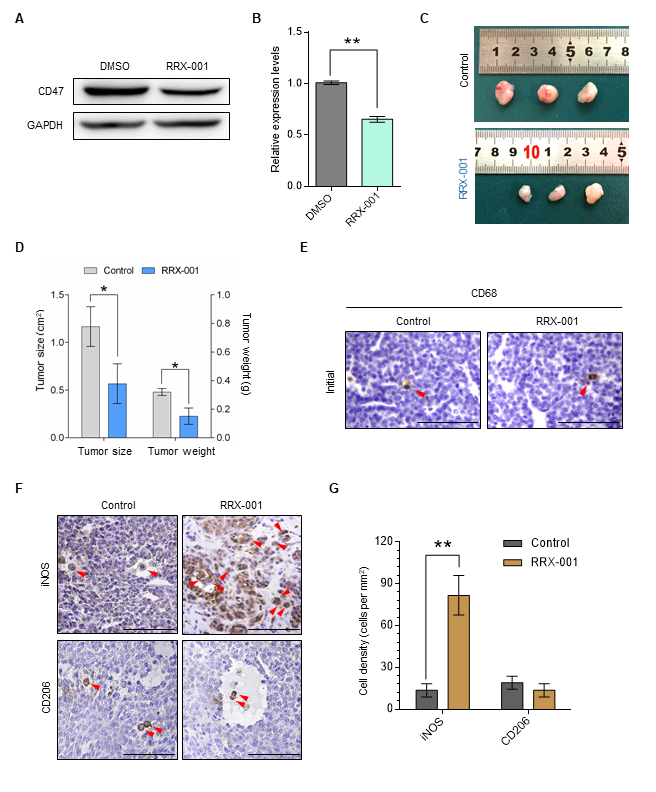
**

**Supplementary Fig. S5.** (related to Figure 6) **RRX-001 skews macrophage differentiation and stimulate phagocytosis in GCTs. A-B,** Western blot results of CD47 protein levels in indicated groups, showing the downregulation of CD47 in GCT cells of the RRX-001 treatment group compared to the DMSO treatment group. Data indicate means ± SD, ***p* <0.01, unpaired *t* test. **C,** Representative images of tumors dissected from C57BL/6 mice treated with vehicle and RRX-001, illustrating that the GCTs in the RRX-001-treated group were significantly smaller than those in the control group. **D**, Statistical results of tumor size and tumor weight with or without RRX-001 treatment, indicating the *in vivo* inhibition of *Cd47* results in a reduction of tumor growth after RRX-001 treatment in C57BL/6 mice. Data represent mean ± SD. *P < 0.05, unpaired *t* test. **E**, Immunohistochemical staining of CD68 (arrowheads) in GCTs at the first day of RRX-001 treatment. **F**, Immunochemistry stainings of iNOS (M1 macrophages marker) and CD206 (M2 macrophages marker) (arrowheads) in the transplanted tumors, showing upregulation of iNOS but not CD206 in tissues after RRX-001 treatment. **G,** Statistic results of F**.** Data represent mean ± SD. **P < 0.01, unpaired *t* test.
