# Supplementary material for: Lineage tracing of mutant granulosa cells reveals in vivo protective mechanisms that prevent granulosa cell tumorigenesis
Source: Cell Death Differ. 2023 Feb 23;30(5):1235–46. doi: 10.1038/s41418-023-01132-1 (PMC10154338; doi:10.1038/s41418-023-01132-1)
Supplement: Supplementary file 2 — Supplementary clinical information [file 41418_2023_1132_MOESM2_ESM.docx]

|  | age | pathologic diagnosis | Source(surgical procedures) |
| --- | --- | --- | --- |
| P1# | 35 | adult type granulosa cell tumor | Hysterectomy and bilateral adnexal resection |
| P2# | 47 | adult type granulosa cell tumor | Hysterectomy and bilateral adnexal resection |
| P3# | 34 | adult type granulosa cell tumor | Hysterectomy and bilateral adnexal resection |
| P4# | 57 | adult type granulosa cell tumor | Hysterectomy and bilateral adnexal resection |
| P5# | 72 | adult type granulosa cell tumor | Hysterectomy and bilateral adnexal resection |
| P6# | 51 | adult type granulosa cell tumor | Hysterectomy and bilateral adnexal resection |
| P7# | 49 | adult type granulosa cell tumor | Hysterectomy and bilateral adnexal resection |
| P8# | 43 | adult type granulosa cell tumor | Hysterectomy and bilateral adnexal resection |
| C1# | 78 | Normal ovarian tissue | Adnexectomy(chronic inflammation of the fallopian tube mucosa) |
| C2# | 73 | Normal ovarian tissue | Adnexectomy(chronic inflammation of the fallopian tube mucosa) |
| C3# | 78 | Normal ovarian tissue | Adnexectomy(chronic inflammation of the fallopian tube mucosa) |
| C4# | 54 | Normal ovarian tissue | Hysterectomy and bilateral adnexal resection(Uterine smooth muscle tumor: adenomyosis) |
| C5# | 53 | Normal ovarian tissue | Hysterectomy and bilateral adnexal resection(Uterine smooth muscle tumor: adenomyosis) |
| C6# | 65 | Normal ovarian tissue | Hysterectomy and bilateral adnexal resection(Uterine smooth muscle tumor) |
| C7# | 72 | Normal ovarian tissue | Hysterectomy and bilateral adnexal resection(Chronic inflammation of the cervical mucosa) |
| C8# | 37 | Normal ovarian tissue | Hysterectomy and bilateral adnexal resection((Right) Adult granulosa cell tumor of the ovary) |

Clinical information for the patient sample included in this paper
