# Supplementary material for: Lineage tracing of mutant granulosa cells reveals in vivo protective mechanisms that prevent granulosa cell tumorigenesis
Source: Cell Death Differ. 2023 Feb 23;30(5):1235–46. doi: 10.1038/s41418-023-01132-1 (PMC10154338; doi:10.1038/s41418-023-01132-1)
Supplement: Supplementary file 3 — Original westernblot images [file 41418_2023_1132_MOESM3_ESM.docx]

Uncropped original western blot result for **Fig 1B**. Immunoblot of PTEN in ovaries of C57 mice on different PDs.

**

**

Uncropped original western blot result for **Fig 1B**. Immunoblot of P27 in ovaries of C57 mice on different PDs.

**

**

Uncropped original western blot result for **Fig 1B**. Immunoblot of ACTINB in ovaries of C57 mice on different PDs.

**

**

Uncropped original western blot result for **Fig 1G**. Immunoblot of PTEN in NoCre control and DKO GCs on PD23.

**

**

Uncropped original western blot result for **Fig 1G**. Immunoblot of P27 in NoCre control and DKO GCs on PD23.

**

**

Uncropped original western blot result for **Fig 1G**. Immunoblot of ACTINB in NoCre control and DKO GCs on PD23.

**

**

Uncropped original western blot result for **Fig 2G**. Immunoblot of PTEN in GCT cells from DKO mice compared to NoCre control mice GCs.

**

**

Uncropped original western blot result for **Fig 2G**. Immunoblot of P27 in GCT cells from DKO mice compared to NoCre control mice GCs.

**

**

Uncropped original western blot result for **Fig 2G**. Immunoblot of ACTINB in GCT cells from DKO mice compared to NoCre control mice GCs.


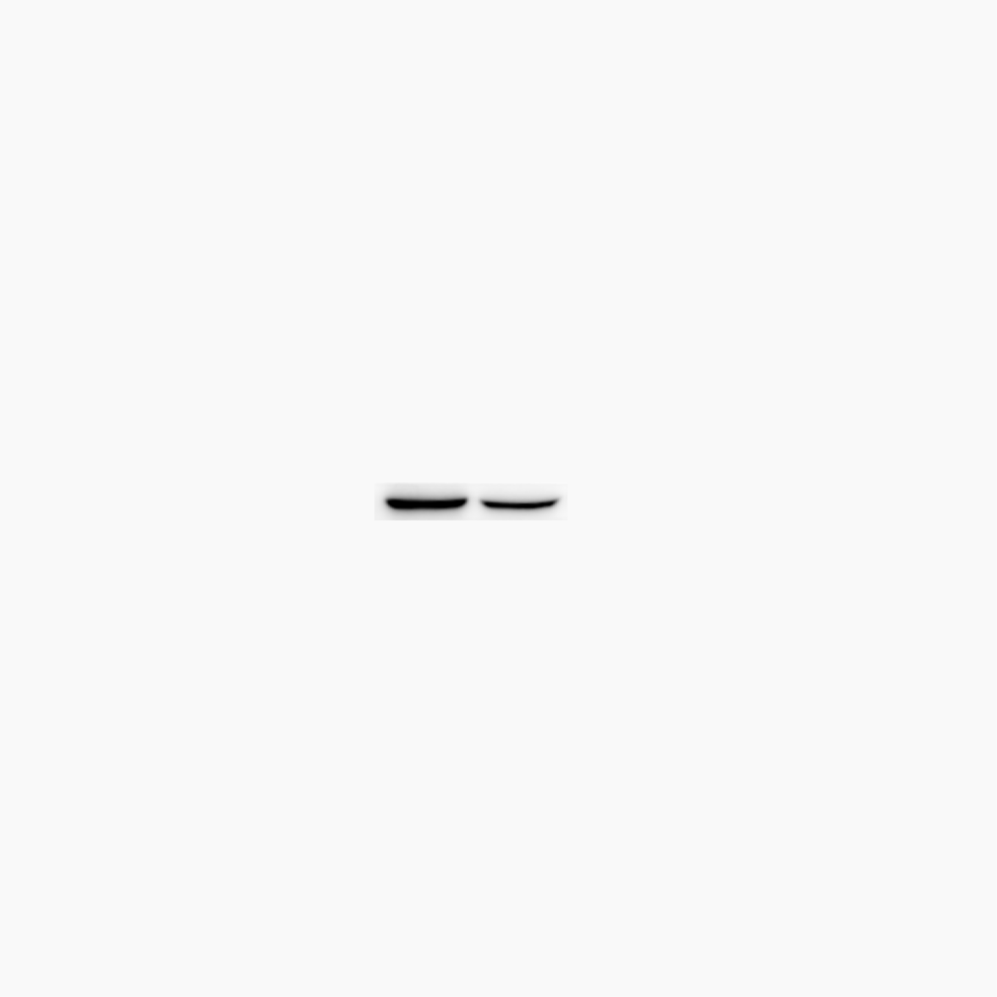


Uncropped original western blot result for **Supplementary Fig S5A**. Immunoblot of CD47 in GCT cells of the RRX-001 treatment group compared to the DMSO treatment group.


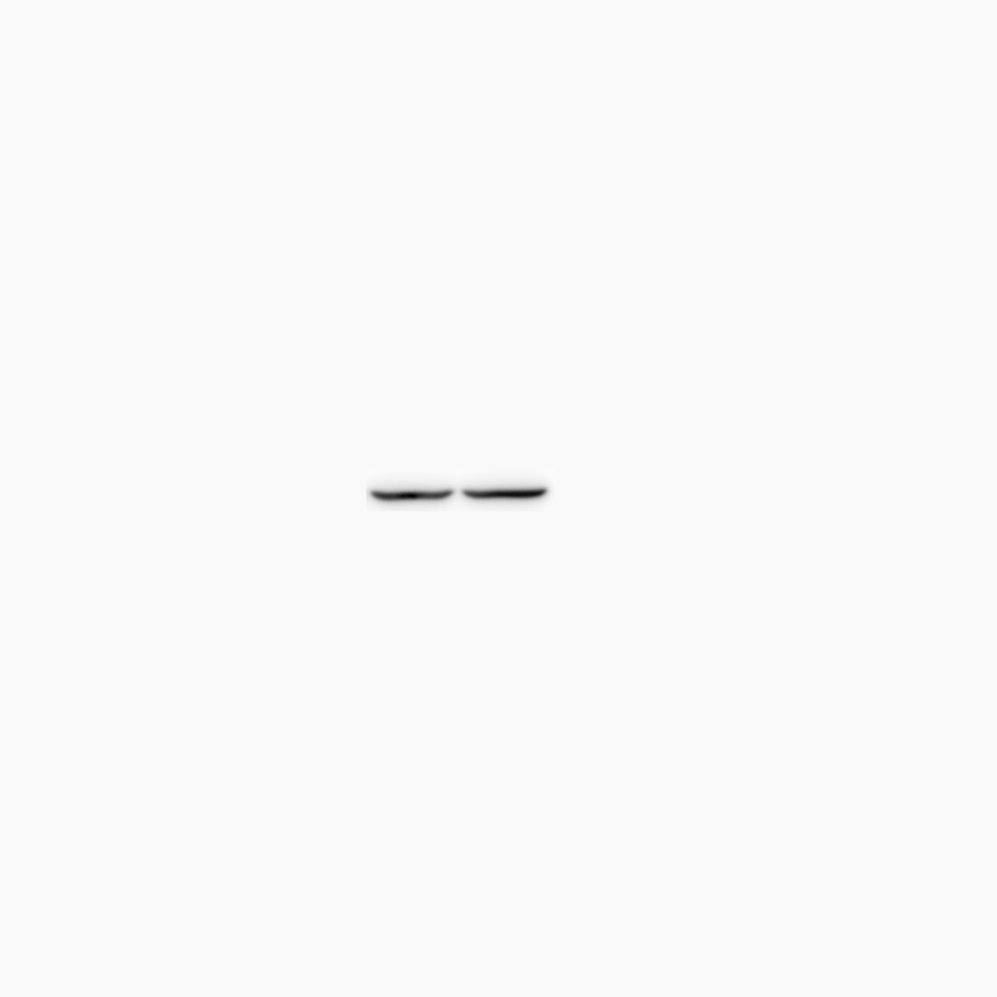


Uncropped original western blot result for **Supplementary Fig S5A**. Immunoblot of GAPDH in GCT cells of the RRX-001 treatment group compared to the DMSO treatment group.
