# Supplementary material for: Lineage tracing of mutant granulosa cells reveals in vivo protective mechanisms that prevent granulosa cell tumorigenesis
Source: Cell Death Differ. 2023 Feb 23;30(5):1235–46. doi: 10.1038/s41418-023-01132-1 (PMC10154338; doi:10.1038/s41418-023-01132-1)
Supplement: Supplementary file 4 — Agreement on author changes [file 41418_2023_1132_MOESM4_ESM.docx]

**Title: Lineage tracing of mutant granulosa cells reveals *in vivo* protective mechanisms that prevent granulosa cell tumorigenesis**

**Ref.:** [**CDD-22-1345RR**](https://mts-cdd.nature.com/cgi-bin/main.plex?form_type=view_ms&j_id=74&ms_id=65700&ms_rev_no=2&ms_id_key=ftdijwAxJw8tNyGYmbouJYKw)

**Author changes declarations**

As request, we have sent email to all co-authors to confirm that they agree to the changes in author list. All of them agree the additions of authors Lijun Huang and Huarong Wang. Co-authors’ email responses are below in this document.

Hua Zhang, Ph.D.

Professor, College of Biological Sciences,

China Agricultural University, China

Feb 8th, 2023


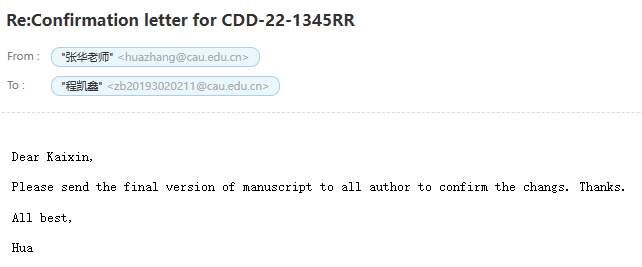


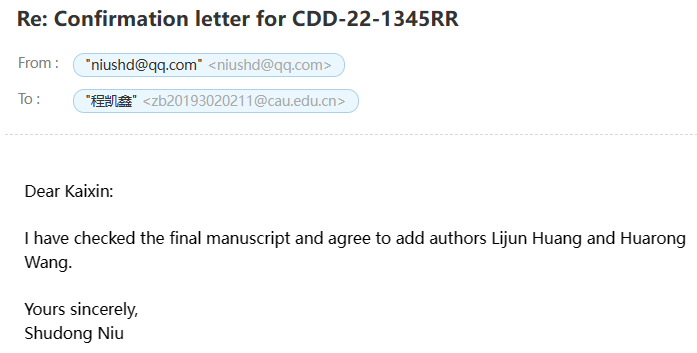


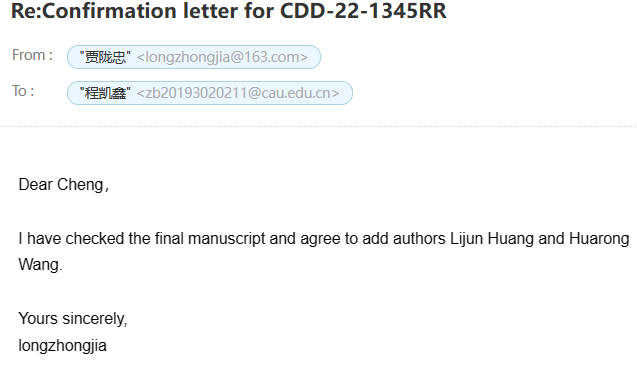


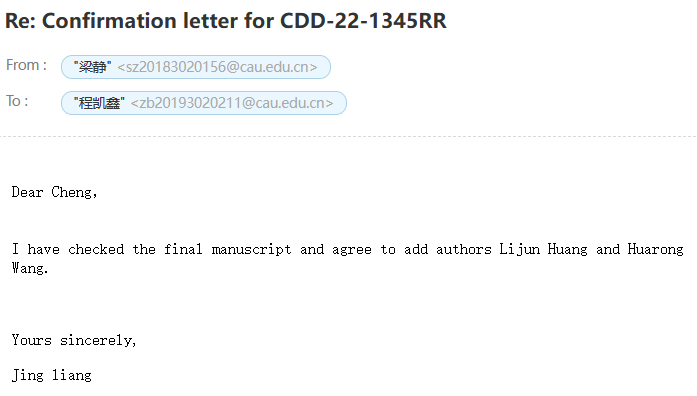


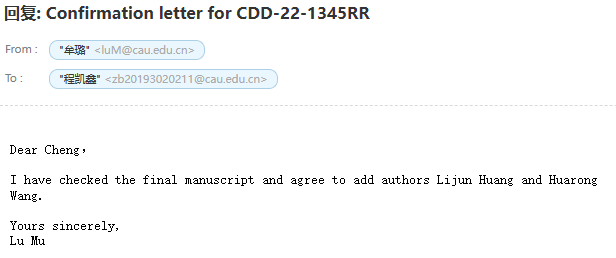


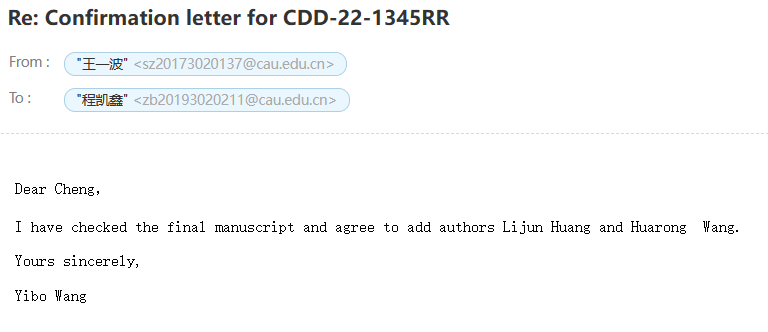


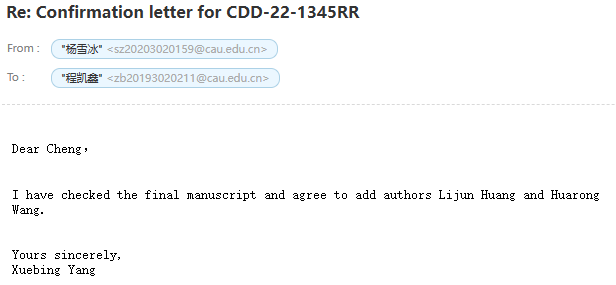


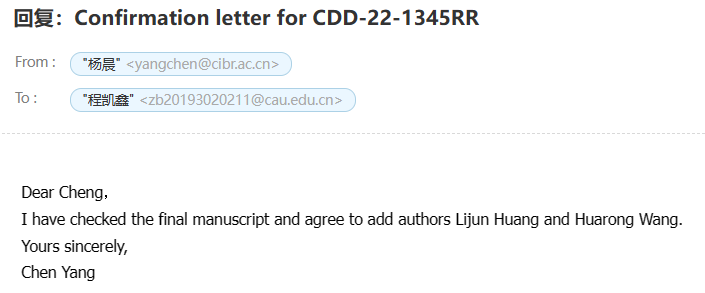


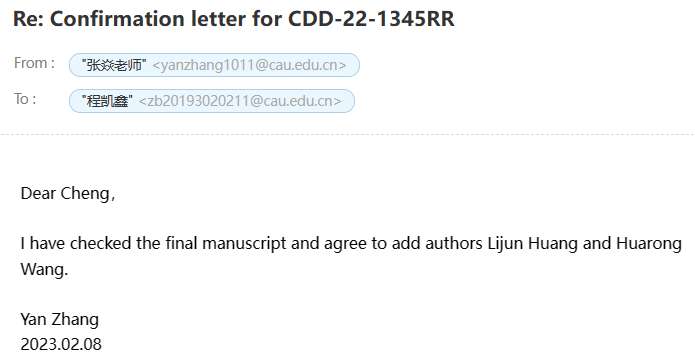


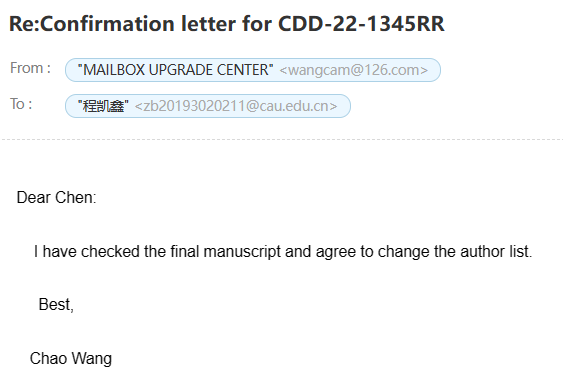


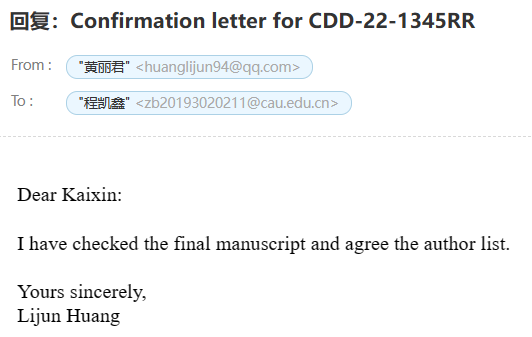


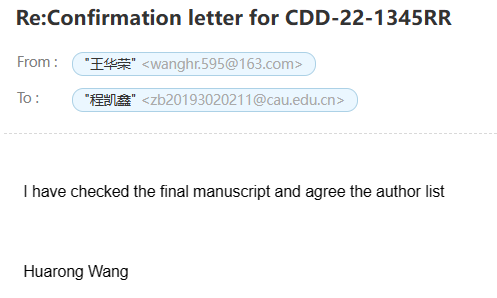


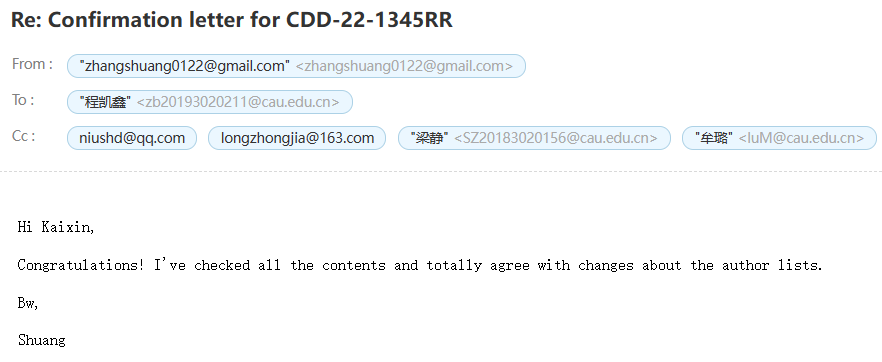


Note: The addressee for these emails is author Kaixin Cheng who also agrees the changes in author list.
